# Fighting Assessment Triggers Rapid Changes in Activity of the Brain Social Decision-Making Network of Cichlid Fish

**DOI:** 10.3389/fnbeh.2019.00229

**Published:** 2019-09-26

**Authors:** Olinda Almeida, Ana S. Félix, Gonçalo A. Oliveira, João S. Lopes, Rui F. Oliveira

**Affiliations:** ^1^ISPA—Instituto Universitário, University Institute of Psychological, Social and Life Sciences, Lisbon, Portugal; ^2^Instituto Gulbenkian de Ciência, Oeiras, Portugal; ^3^Champalimaud Neuroscience Programme, Champalimaud Centre for the Unknown, Lisbon, Portugal

**Keywords:** social decision making network, social competence, immediate early genes, androgens, challenge hypothesis

## Abstract

Social living animals have to adjust their behavior to rapid changes in the social environment. It has been hypothesized that the expression of social behavior is better explained by the activity pattern of a diffuse social decision-making network (SDMN) in the brain than by the activity of a single brain region. In this study, we tested the hypothesis that it is the assessment that individuals make of the outcome of the fights, rather than the expression of aggressive behavior *per se*, that triggers changes in the pattern of activation of the SDMN which are reflected in socially driven behavioral profiles (e.g., dominant vs. subordinate specific behaviors). For this purpose, we manipulated the perception of the outcome of an agonistic interaction in an African cichlid fish (*Oreochromis mossambicus*) and assessed if either the perception of outcome or fighting by itself was sufficient to trigger rapid changes in the activity of the SDMN. We have used the expression of immediate early genes (*c-fos* and *egr-1*) as a proxy to measure the neuronal activity in the brain. Fish fought their own image on a mirror for 15 min after which they were allocated to one of three conditions for the two last minutes of the trial: (1) they remained fighting the mirror image (no outcome treatment); (2) the mirror was lifted and a dominant male that had just won a fight was presented behind a transparent partition (perception of defeat treatment); and (3) the mirror was lifted and a subordinate male that had just lost a fight was presented behind a transparent partition (perception of victory treatment). Results show that these short-term social interactions elicit distinct patterns in the SDMN and that the perception of the outcome was not a necessary condition to trigger a SDMN response as evidenced in the second treatment (perception of defeat treatment). We suggest that the mutual assessment of relative fighting behavior drives these acute changes in the state of the SDMN.

## Introduction

Individuals from social species need to combine information about the social environment they live in with information about their internal state, such as previous social experience and organismal condition, in order to adaptively optimize their responses to changes in the social environment (Taborsky and Oliveira, 2012). This ability to rapidly and adaptively adjust behavior to daily social demands is known as social competence and is thought to be accomplished through rapid changes in the state of the neural network underlying social behavior (Oliveira, 2012). Accordingly, consistent changes in social behavior, such as adopting a dominant or subordinate behavioral profile, are associated with distinct behavioral states (that express different behavioral patterns) that are paralleled by specific states of the social-decision making network (SDMN) in the brain (Cardoso et al., 2015). The SDMN consists of an evolutionarily conserved set of core brain nuclei that together regulate the expression social behavior, such that the state of the network better explains the behavioral output rather than the activity of a single node *per se* (Goodson, 2005; Newman, 1999; O’Connell and Hofmann, 2011b, 2012). All of these brain nuclei are reciprocally interconnected with each other, such that differential activation of the nodes creates dynamic patterns responsible for multiple behaviors. Moreover, the nodes of the SDMN have an extensive expression of steroid neuropeptide and aminergic receptors, which allows this network to be modulated by these hormones, probably by altering the weight of its nodes or the strength of their connectivity (Goodson, 2005; O’Connell et al., 2011; O’Connell and Hofmann, 2011a; Oliveira, 2012). Thus, different behavioral states should result from divergent transcriptomes of the SDMN, and changes between states, such as acquiring or losing social status should be associated with rapid changes in patterns of gene expression in the SDMN. Given their fast and transient response to changes in extra- and intra-cellular environment and their effect as transcription factors, immediate early genes (e.g., *c-fos, egr-1*) play a key role in orchestrating transcriptomic responses to environmental changes. Thus, it has been hypothesized that immediate early genes can be the molecular first responders to perceived changes in the social environment that trigger subsequent changes in the neurogenomic state of the SDMN that allows the animal to adjust its behavioral state accordingly (Cardoso et al., 2015). Several studies have documented changes in immediate early gene (IEG) expression across the SDMN associated with changes in social behavior across different vertebrate taxa (e.g., Faykoo-Martinez et al., 2018; Kabelik et al., 2018; O’Connell and Hofmann, 2012), including teleost fish and also tilapia (e.g., Field and Maruska, 2017; Roleira et al., 2017; Teles et al., 2015). In particular, changes in social status (i.e., ascending or descending in a social hierarchy) have been associated with rapid changes in IEG expression in the SDMN paralleled by changes in social behavior (Maruska et al., 2013a,b; Teles et al., 2015;Williamson et al., 2019).

In this study, we sought to understand what are the key aspects of an agonistic interaction that trigger an IEG response across the SDMN and concomitantly a socially driven neuromolecular restructuring of this network. We reasoned that in order to be adaptive such network restructuring should match the post-fight social scenario anticipated by the individual in face of the information collected during the interaction. Therefore, the perception of the fight outcome rather than the expression of aggressive behavior *per se* should play a key role in triggering the SDMN IEG response to an aggressive interaction. Here, we have tested if the perception of the outcome of a single agonistic interaction in an African cichlid fish (*Mozambique tilapia*, *Oreochromis mossambicus*) is necessary to trigger an IEG response across the SDMN or if fighting itself is sufficient to trigger the response.

In order to manipulate the perception of fight outcome, we took advantage of the fact that male Tilapia do not recognize their own image in a mirror and fight aggressively towards it (e.g., Oliveira et al., 2005; Teles et al., 2013). Because in mirror fights the opponent’s behavior (i.e., mirror image) always matches the behavior of the focal fish, there is no information available to the participant regarding the fight outcome. That is the males express aggressive behavior without experiencing either a win or a defeat. Thus, an IEG response triggered by a mirror fight would be driven by the experience of fighting and not by the perception of the interaction outcome (i.e., winning vs. losing). In this study, we have used three fighting treatments. After a mirror fighting phase that lasted 15 min focal males were allocated to one of three conditions for the last 2 min of the trial: (1) they remained fighting their mirror image (no outcome treatment, where the mirror image remained in both steps of the experiment; MM); (2) the mirror was lifted and a dominant male that had just won a fight was presented behind a transparent partition (opponent becoming dominant treatment, where the mirror image became dominant male; MD); and (3) the mirror was lifted and a subordinate male that had just lost a fight was presented behind a transparent partition (opponent becoming subordinate treatment, where the mirror image became a subordinate male; MS). Our prediction was that if the IEG response is challenge dependent, then all three treatments would trigger a similar IEG response; in contrast, if IEG responsiveness is dependent on perceiving a win or a defeat, divergent IEG responses across the SDMN are expected in the MD and MS treatments in relation to the mirror fights treatment (MM) where no information on outcome is available. Given that socially-driven changes in the SDMN are expected to produce integrated phenotypic responses, at the behavioral and physiological (hormonal) levels, to the social environment and that androgens have been described to respond to social challenges (challenge hypothesis, Hirschenhauser and Oliveira, 2006; Wingfield et al., 1990), we have also characterized the response of the hypothalamic-pituitary-gonadal (HPG) axis to our experimental treatments by measuring the expression of gonadotrophin-releasing hormone (*gnrh1*) in the preoptic area and circulating androgen levels (testosterone, T, and 11-ketotestosterone, KT).

## Materials and Methods

### Animals and Housing

The *Mozambique tilapia* is a freshwater fish with a lek-breeding system (Fryer and Iles, 1972). Males aggregate densely in mating territories, where they dig and defend spawning pits and compete for females (Oliveira and Almada, 1998). Males present two distinct phenotypes, which can rapidly reverse due to changes in the social environment (Oliveira and Almada, 1998). Dominant males are usually larger, dark-colored, establish territories and attract females. In contrast, subordinate males have a silver color pattern similar to that of females, and fail to establish territories.

*O. mossambicus* fish from a stock held at ISPA was used in this study. Fish were maintained in stable social groups of four males and five females per group, in glass tanks (120 × 40 × 50 cm, 240 L) with a fine gravel substrate. Tanks were supplied with a double filtering system (sand and external biofilter; Eheim) and constant aeration. Water quality was monitored on a weekly basis for nitrite (0.2–0.5 ppm), ammonia (<0.5 ppm; Pallintest kit) and pH (6.0–6.2). Fish were kept at a temperature of 26 ± 2°C, a 12L:12D photoperiod, and fed with commercial cichlid sticks. The social status of the males was monitored daily and territorial males were identified by dark body coloration and digging of a spawning pit on the substrate (Oliveira and Almada, 1996).

### Experimental Procedure

**Figure 1:**
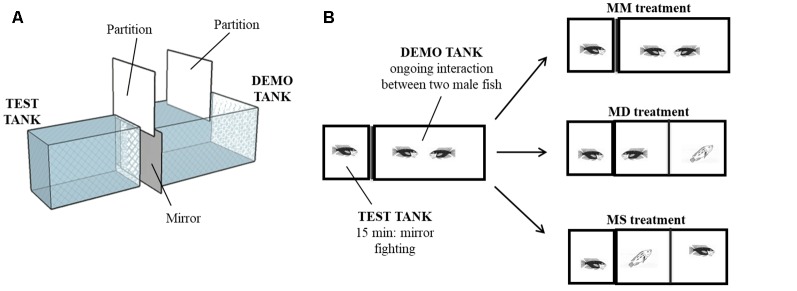
Behavioral paradigm. **(A)** 3D diagram of the experimental setup. Test tank and demo tank were side-by-side and physically separated. **(B)** Schematic of the experimental treatments. Focal fish interacted with a mirror for 15 min while two males were fighting in the adjacent compartment. Then, focal fish were allowed to see for 2 min its own image in the mirror (MM treatment), a dominant male (Mirror becomes Dominant—MD treatment) or a subordinate male (Mirror becomes Subordinate—MS treatment).

The experimental setup consisted of two adjacent tanks (test and demo tank) with an opaque partition between them. Twenty territorial focal males (mean body mass ± SEM: 81.63 g ± 7.06 g) were used in this experiment. Each focal male was isolated for 7 days in the test tank (30 × 50 × 25 cm). On day 6, plasma was collected from the focal male to determine steroids baseline levels. On the same day, a male fish was introduced in the demo tank (30 ×70 × 40 cm), to allow it to adopt this tank as its territory. On the day of the experiment (day 7), an intruder male was introduced in the demo tank and both males were allowed to interact for 30 min. This agonistic interaction was accompanied by the experimenter and fight outcome was assessed by live observation. Accordingly, after fight resolution, winners continue to be aggressive and present a dark coloration while losers only display submissive behavior and present a light coloration. Thus, winners can be seen as clear/explicit dominant males (recently gaining social status) and losers as clear subordinate males (recently losing social status). Fifteen minutes after the beginning of the social interaction in the demo tank, a mirror was placed in the external wall of the test tank, adjacent to the demo tank. The interaction between the mirror and the focal male in the test tank was recorded for 15 min. At the end of the mirror interaction, males in the demo tank were separated by an opaque partition and the focal male in the test tank was allowed to see for 2 min one of the following stimuli: (i) its own image in the mirror (MM treatment, *N* = 8), or a real (opponent) male, either; (ii) the dominant male of the demo tank (Mirror becomes Dominant—MD treatment, *N* = 6); or (iii) the subordinate male of the demo tank (Mirror becomes Subordinate—MS treatment, *N* = 6; Figure 1). Fight outcome was manipulated by controlling the order of introduction of each fish in the demo tank and their size, so the male introduced first (in day 6) was always bigger than the intruder and won all staged fights. Using this procedure, we had no unsolved fights. Focal and opponent males were sized matched and were selected from different family tanks to control for familiarity effects. At the end of the experiment, an opaque partition was placed between the tanks to prevent the males from seeing each other and 20 min later a blood sample was collected from the caudal vein under anesthesia (MS-222, Pharmaq; 300–400 ppm). Blood sampling always took less than 4 min from the induction of anesthesia to prevent possible effects of handling stress on steroids levels (Foo and Lam, 1993). Blood samples were centrifuged (10 min, 600 *g*) and plasma was stored at −20°C until further processing. After blood sampling, the fish were returned to the anesthesia solution until muscular and opercular movements stopped completely and were then sacrificed by decapitation. The cranial fraction (brain and part of the cranial bones) was embedded in mounting media (OCT Compound, Tissue-Tek, Sakura) and frozen at −80°C during 15–30 min. Coronal sections were obtained at 150 μm thickness using a cryostat (Microm HM 500 M) and collected on previously cleaned slides (70% ethanol). Regions of interest were microdissected under a steromicroscope (VWR SZB350OH) and collected in 50 μl of Qiazol lysis buffer (RNeasy Lipid Tissue Mini Kit, Qiagen) with a modified 25G needle. Samples were stored at −80°C until RNA extraction. The following representative nodes of the SDMN (O’Connell and Hofmann, 2011b) were identified according to Teles et al. (2012): medial part of the ventral subdivision of the ventral telencephalon (VVm; putative homolog of the mammalian lateral septum), supracommissural part of the ventral telencephalon (Vs; putative homolog of the mammalian medial extended amygdala), anterior part of the periventricular preoptic nucleus (PPa), nucleus anterior tuberis (TA; putative homolog of the ventromedial hypothalamus) and central gray (GC).

### Behavioral Observations

The behavior of the focal male, either towards the mirror or interacting with the opponent male, was analyzed using a computerized multi-event recorder software (Observer, Noldus technology, version 5). The behavior of the opponent male was also analyzed with the same software (see Supplementary Figure S1 for the descriptive statistics of focal and opponent behavioral measures). The analysis was based on the ethogram repertoire provided by Baerends and Baerends-Van Roon (1950). Relevant behavioral patterns were identified to measure male aggressive behavior (i.e., bites, displays, attacks).

### Gene Expression Analysis

Primers were designed using National Center for Biotechnology Information (NCBI) sequences for *c-fos* (accession #GR607679.1), *egr-1* (accession #AY493348.1), *gnrh1* (accession #AB101665.1) and the housekeeping gene *eef1A* (accession #AB075952.1). Primer3 software (Koressaar and Remm, 2007; Untergasser et al., 2012) was used to design the primers, which were commercially synthesized (Sigma-Aldrich, Hamburg, German). Primers were tested with a cDNA pool in a qRT-PCR, and PCR products were confirmed by sequencing. Amplification products were 106 pb for *c-fos*, 135 pb for *egr-1*, 127 pb for *gnrh1* and 85 pb for *eef1A*. Primer dimer formation was controlled with FastPCR v5.4 software (Kalendar et al., 2017) and optimal annealing temperature was assessed for maximal fluorescence (Supplementary Table S1). qRT- PCR was performed using the Quantitative PCR System Stratagene MX3000P. The reaction mix included Sybr Green (Fermentas, #K0221), 400 nM of each primer and 1 μl of cDNA in a 25 μl reaction volume. Cycling parameters were: (i) denaturation: 5 min at 95°C; (ii) amplification and quantification: 40 cycles (30 s at 95°C, 30 s at primer-specific annealing temperature, 30 s at 72°C); and (iii) dissociation curve assessment (30 s at 95°C, 30 s at 55°C, 30 s at 95°C). The dissociation curve was performed to confirm a single melting curve proving the inexistence of primer-dimer formation and/or plate contamination. All samples were run in triplicate and controls with water instead of DNA templates showed no amplification. PCR Miner (Zhao and Fernald, 2005) was used to calculate reaction efficiencies (E) and cycle thresholds (CT), based on the kinetics of individual PCR reactions. *c-fos*, *egr-1* and *gnrh1* mRNA levels normalized for housekeeping (HK) gene *eef1A* were determined from the equation: ${\left(1+E_{HK}\right)}^{CT_{HK}}/{\left(1+E_{\text{gene}}\right)}^{CT_{\text{gene}}}$. Mean values for eef1A did not differ between treatments, thus confirming its suitability to be used as a reference gene in this study.

### Quantification of Steroids Levels

Free steroids (testosterone, T; and 11-ketotestosterone, KT) were extracted from plasma samples by adding diethyl-ether to the samples, centrifuging the mix (800 *g*, 5 min, 4°C) and freezing it (15 min, −80°C) to separate the ether fraction (containing the free steroid). This process was repeated twice. The ether fraction was evaporated and the steroids were re-suspended in phosphate buffer. Steroid concentrations were measured by radioimmunoassay. The testosterone antibody was from Research Diagnostics Incorporation (#WLI-T3003, rabbit anti-testosterone) and the 11-ketotestosterone antibody was kindly donated by D. E. Kime (the specificity table was published in Kime and Manning, 1982). We used a testosterone reactive marker from Amersham Biosciences [(1, 2, 6, 7–3H) testosterone, #TRK402-250 μCi] and a titrated 11-ketotestosterone produced in-house from marked cortisol (Kime and Manning, 1982). Inter-assay variabilities were 4.1% and 8.9% for T and KT, respectively. Intra-assay variation coefficients were 2.4% and 2.0% for T and 4.1% and 4.0% for KT.

### Data Analysis

Outlier observations were identified and replaced by missing values using a generalized extreme studentized deviate procedure (e.g., Jain, 2010) with a *p*-value of 0.05 and a maximum number of outliers set at 20% of the sample size. Behavioral variables and gene expression levels were logarithmically transformed [log10 (x + 1)] to meet parametric test assumptions. The behavioral variables (for frequency and latency) were reduced with Principal Component Analysis (PCA) using the variable principle normalization method. Two principal components (PC) were obtained that explain 86.3% of the variance and that seem to represent different aspects of aggressive behavior: “overt aggression” and “aggressive motivation” (see “Results” section). The component scores of each case on each of these PC were analyzed using separate Linear Mixed Models (LMM) with Treatment (MM, MD, MS) as a fixed effect and focal fish as a random effect. *Post hoc* tests were used to test for differences between experimental treatments, with *p*-values adjusted for the number of multiple comparisons (Benjamini and Hochberg, 1995).

Separate LMM were also used to check for differences between treatments in IEG (*c-fos, egr-1*) expression in each sampled brain area (GC, TA, Vs, VVm, PPa). *Post hoc* tests were used to test for differences between experimental treatments, with *p*-values adjusted for the number of multiple comparisons (Benjamini and Hochberg, 1995).

Pearson correlations between IEG expression of each brain area and between the behavioral principal component score were used to examine the association between aggressive behavior and gene expression. Pearson correlation matrices between each pair of brain nuclei for each IEG were used as a measure of functional connectivity and tested using a Quadratic Assignment Procedure (QAP) with 5,000 permutations. Since the null-hypothesis for QAP states that there is a non-random association between the tested matrices, a QAP with a non-significant *p*-value indicates that there is no association between the treatment’s IEG activational pattern. The *p*-values of the Pearson correlation matrices were adjusted (Benjamini and Hochberg, 1995). The brain patterns of IEG expression obtained for each experimental treatment were tested on a network perspective, by measuring density and centrality parameters (Makagon et al., 2012). Density was used as a measure of the network cohesion, given by the proportion of all possible connections that are present in the network (Makagon et al., 2012). Differences in network density between treatments were tested using a *t*-test (bootstrap set to 5,000 sub-samples). As a measure of node centrality we assessed eigenvector centrality, that takes into account not only how well a node is connected to other nodes in the network but also how well connected its relations are (Makagon et al., 2012).

Variation in hormone levels (KT, T) was computed as (Post-treatment levels) − (Baseline levels) for each individual. To test for differences between the treatments we performed unpaired *t*-tests. Pearson correlation analysis was used to examine the relationship between *gnrh1* gene expression and IEG expression in the PPa. Pearson correlation analysis was also used to examine the relationship between *gnrh1* gene expression in the PPa and androgen circulating levels. A LMM was used to test for differences between treatments in *gnrh1* in the PPa area. *Post hoc* tests were used to test for differences between experimental treatments, with *p*-values adjusted for the number of multiple comparisons (Benjamini and Hochberg, 1995).

Effect sizes were computed for *post hoc* tests (Cohen’s *d*).

Statistical analysis was performed using IBM SPSS^®^ statistics v.21, and R (R Core Team, 2015) with the following packages: nlme (LMM), dplyr (*t*-tests), multcomp (*post hoc* comparisons), Hmisc (correlations), ggplots (heatmaps). Characterization of the SDMN network was obtained with UCINET version 6.653 (Borgatti et al., 2002). Brain nuclei representations of the SDMN network were produced using a custom-made python script. Degrees of freedom may vary between the analyses due to missing values.

### Ethics Statement

In this study, we have staged real opponent agonistic interactions to obtain winner and loser animals, since the use of video-playbacks in this species is inadequate (RO, personal observation). However, we have kept sample sizes to a minimum, and limited contests to a short duration. No signs of physical injuries were observed during any of the trials. Animal experimentation procedures were conducted in accordance with the European Communities Council Directive of 24 November 1986(86/609/EEC) and were approved by the Portuguese Veterinary Authority (Direcção Geral de Alimentação e Veterinária, Portugal; permit # 0421/000/000/2013).

## Results

### Behavior

A PCA of the behavioral variables resulted in two PC that together explained 86.3% of the variance in aggressive behavior (Table 1). PC1 had a high loading (>0.9) of frequency of bites and frequency of attacks, and hence it was interpreted as “overt aggression.” The highest loading in PC2 was the latency to display, and hence its symmetric was interpreted as “aggressive motivation.”

**Table 1:** Principal component analysis (PCA) of behavioral variables.

Behavioral variables	Component loading	
	PC1	PC2
Frequency of displays	0.793	−0.443
Frequency of bites	0.915	−0.161
Frequency of attacks	0.923	0.122
Latency to display	−0.595	0.717
Latency to bite	−0.887	−0.293
Latency to attack	−0.896	−0.287
Eigenvalue	4.262	0.919
% of variance explained	71.03	15.32

**Figure 2:**
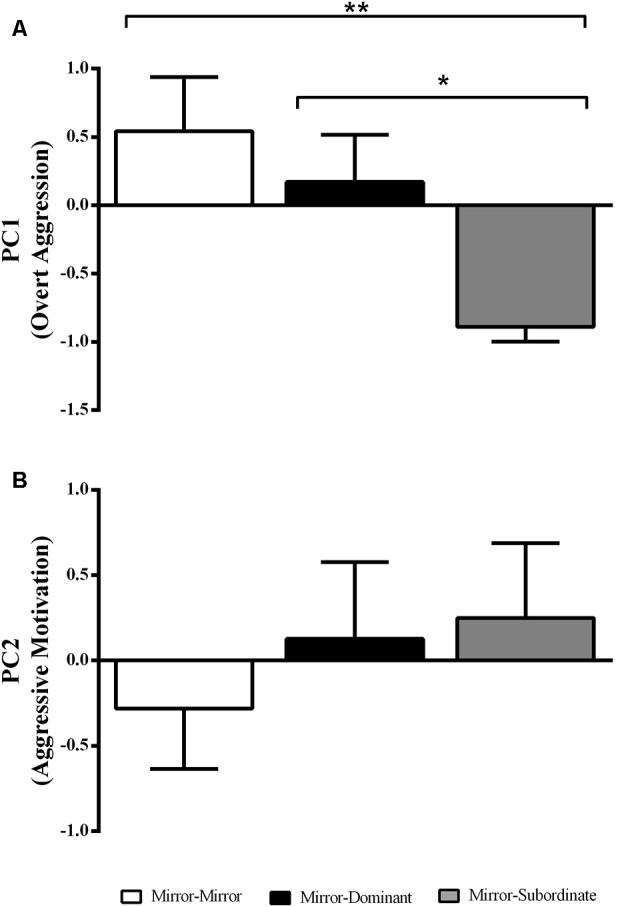
Variation in the behavioral component scores obtained with the Principal Component Analysis (PCA) for each experimental treatment. **(A)** PC1 interpreted as “overt aggression”; and **(B)** PC2 interpreted as “aggressive motivation.” *Significant difference for *p* < 0.05; **significant difference for *p* < 0.01. Results are expressed as mean ± standard error of the mean (SEM).

There was an effect of the experimental treatment in “overt aggression” (i.e., PC1 loadings; *F*_(2,17)_ = 4.87, *p* = 0.02), with focal fish assigned to the MS condition showing significantly less overt aggression than those in the MM and MD conditions (Figure 2A). In contrast there was no effect of experimental treatment on “aggressive motivation” (PC2 loadings; *F*_(2,17)_ = 0.50, *p* = 0.62; Figure 2B).

### Immediate Early Gene Expression in the Social Decision-Making Network (SDMN)

**Figure 3:**
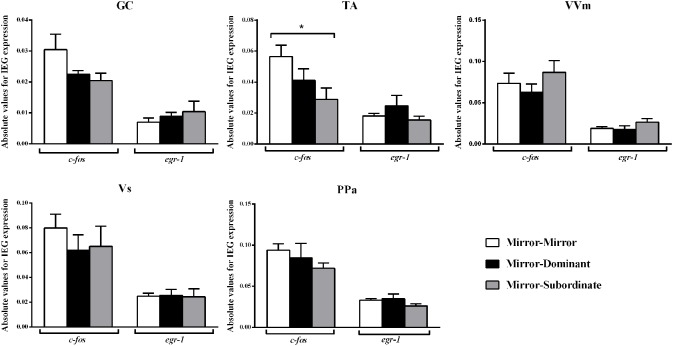
Expression of the immediate early genes (IEG) *c-fos* and *egr-1* in several brain areas of the social decision-making network (SDMN). GC, central gray; PPa, anterior part of the periventricular preoptic nucleus; TA, nucleus anterior tuberis; VVm, medial part of the ventral subdivision of the ventral telencephalon; Vs, supracommissural nucleus of the ventral telencephalon. *Significant difference for *p* < 0.05. Results are expressed as mean ± standard error of the mean (SEM).

Significant differences between treatments were only detected for *c-fos* in the TA area, specifically between the MM and the MS treatments (Figure 3; Table 2). No other significant main effect or *post hoc* comparison was detected for *c-fos* or *egr-1*.

**Table 2:** Effect of treatment on immediate early genes expression in social decision-making network (SDMN) areas.

	Main effects		MM vs. MD			MM vs. MS			MD vs. MS		
Areas	*F*	*p*	*t*	*p*	*d*	*t*	*p*	*d*	*t*	*p*	*d*
c-fos										
VVm	0.816	0.462	0.550	0.583	0.031	0.820	0.583	0.050	1.268	0.583	0.086
Vs	0.821	0.458	1.004	0.473	0.072	1.170	0.473	0.061	0.160	0.873	0.008
TA	3.839	**0.042**	1.250	0.211	0.081	2.770	**0.017**	0.140	1.421	0.211	0.069
GC	0.426	0.663	0.910	0.363	0.091	0.319	0.750	0.017	0.591	0.555	0.036
PPa	0.970	0.400	1.027	0.457	0.047	1.277	0.457	0.119	0.286	0.775	0.016
*egr-1*											
VVm	1.528	0.247	0.675	0.500	0.038	1.119	0.395	0.070	1.729	0.252	0.087
Vs	0.156	0.857	0.166	0.868	0.010	0.552	0.868	0.030	0.362	0.868	0.018
TA	1.176	0.333	0.808	0.419	0.040	0.831	0.419	0.057	0.1.533	0.376	0.074
GC	0.918	0.419	1.094	0.411	0.066	1.174	0.411	0.059	0.130	0.897	0.008
PPa	1.705	0.213	0.078	0.938	0.004	1.600	0.164	0.109	1.618	0.164	0.081

*Main effects and post hoc comparisons between treatments. d: effect size estimate (Cohen’s d); Treatments: MM, Mirror-Mirror; MD, Mirror-Dominant; MS, Mirror-Subordinate; GC, central gray; PPa, anterior part of the periventricular preoptic nucleus; TA, nucleus anterior tuberis; VVm, medial part of the ventral subdivision of the ventral telencephalon; Vs, supracommissural nucleus of the ventral telencephalon; *c-fos* degrees of freedom for F-test: GC: (2, 12); PPa: (2, 16); TA: (2, 17); VVm: (2, 14); Vs: (2, 16); *egr-1* degrees of freedom for F-test: GC: (2, 16); PPa: (2, 16); TA: (2, 17); VVm: (2, 16); Vs: (2, 17); statistically significant values are in bold*.

No significant association between the correlation matrices for *c-fos* and *egr-1* expression in the brain areas of the SDMN was detected using QAP, suggesting that all treatments showed a distinct co-activation pattern for *c-fos* and *egr-1* (Table 3, Supplementary Figure S2). Thus, the pattern of functional connectivity across the SDMN is specific for each treatment. The density of the *egr-1* network was significantly higher for fish assigned to the MS treatment when compared to the MM and MD treatments (MM vs. MS: *t* = 2.815, *p* = 0.005; MD vs. MS: *t* = 2.061, *p* = 0.037; Table 4). The *egr-1* network density for MM and MD treatments was not significantly different (MM vs. MD: *t* = 1.488, *p* = 0.137). We have not detected significant differences between treatments for *c-fos* network density (MM vs. MD: *t* = 1.861, *p* = 0.065; MM vs. MS: *t* = 0.461, *p* = 0.607; MD vs. MS: *t* = 1.588, *p* = 0.125). The eigenvector centrality measures suggest that GC is a central node in the *c-fos* and *egr-1* networks for fish in the MM and MS treatments, but that it is a poorly connected node in the MD treatment (Table 4). The eigenvector centrality measures show that the MD and MS treatment networks are characterized by a high centrality of the PPa node for *egr-1* (Table 4). Centrality measures of the *egr-1* network for fish in the MM treatment show a high centrality for TA and a low centrality for PPa (Table 4).

**Table 3:** Association between the correlation matrices for immediate early gene (IEG) expression in the brain areas of the SDMN.

		MM		MD	
		*r*	*p*	*r*	*p*
c-fos	MS	−0.202	0.291	−0.119	0.409
	MD	0.148	0.367		
*egr-1*	MS	−0.222	0.259	−0.134	0.501
	MD	−0.489	0.189		

*Quadratic assignment procedure (QAP) for *c-fos* and *egr-1* co-activation matrices. Treatments: MM, Mirror-Mirror; MD, Mirror-Dominant; MS, Mirror-Subordinate*.

**Table 4:** Characterization of the SDMN for each experimental treatment using *c-fos* and *egr-1* as reporters of neuronal activity.

		*c-fos*			*egr-1*		
		MM	MD	MS	MM	MD	MS
Density		0.559	0.360	0.535	0.243	0.391	0.553
eigenvector	GC	0.550	0.175	0.565	0.532	0.459	0.542
	PPa	0.408	0.579	0.382	0.127	0.576	0.518
	TA	0.455	0.264	0.398	0.644	0.374	0.380
	VVm	0.456	0.523	0.375	0.454	0.188	0.444
	Vs	0.342	0.538	0.486	0.282	0.532	0.310

*Values reported correspond to network cohesion (density) and centrality (eigenvector) of each node of the network. Treatments: MM, Mirror-Mirror; MD, Mirror-Dominant; MS, Mirror-Subordinate; GC, central gray; PPa, anterior part of the periventricular preoptic nucleus; TA, nucleus anterior tuberis; VVm, medial part of the ventral subdivision of the ventral telencephalon; Vs, supracommissural nucleus of the ventral telencephalon*.

**Figure 4:**
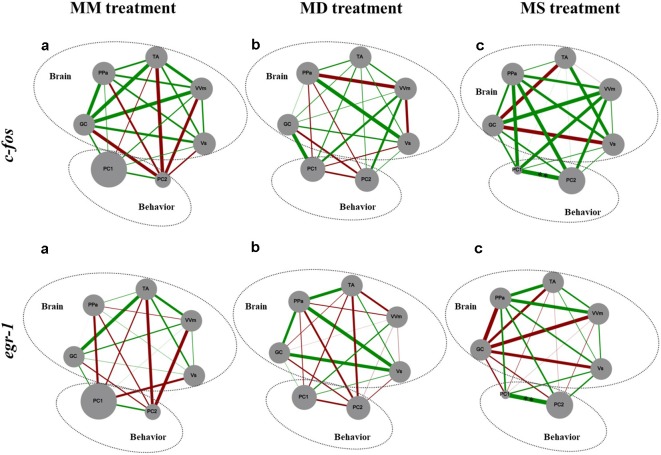
Representation of the state of the SDMN and the behavior for all the experimental treatments. Node size of each brain area indicates the activity level at each network node using *c-fos* and *egr-1* as reporters of neural activity. PC1 and PC2, component loadings obtained with the PCA of aggressive behavior were used as behavioral network nodes, where the node size corresponds to the average of principal component scores within each treatment. Line thickness indicates the strength of the connection between nodes (measured with Pearson correlation coefficients, *r*-value); green lines represent positive correlations; red lines represent negative correlations. GC, central gray; PPa, anterior part of the periventricular preoptic nucleus; TA, nucleus anterior tuberis; VVm, medial part of the ventral subdivision of the ventral telencephalon; Vs, supracommissural nucleus of the ventral telencephalon. PC1, first component loading interpreted as “overt aggression”; PC2, second component loading interpreted as “aggressive motivation.” **Significant correlations after *p*-value adjustment for *p* < 0.01.

There were no significant correlations between *c-fos* or *egr-1* expression in brain areas of the SDMN and aggressive behavior (Figure 4).

### Activity of the Hypothalamic-Pituitary-Gonadal (HPG) Axis

There were no significant correlations between the neuronal activation of the PPa as measured by either *c-fos* or *egr-1* and the expression of gnrh1 in the PPa or circulating androgen levels (*c-fos*: *r* = 0.170, *p* = 0.499, *n* = 18; *egr-1*: *r* = 0.107, *p* = 0.673, *n* = 18). There were also no significant correlations between the expression of *gnrh1* in the PPa and circulating androgen levels (KT: *r* = 0.276, *p* = 0.283, *n* = 17; T: *r* = 0.371, *p* = 0.143, *n* = 17).

**Figure 5:**
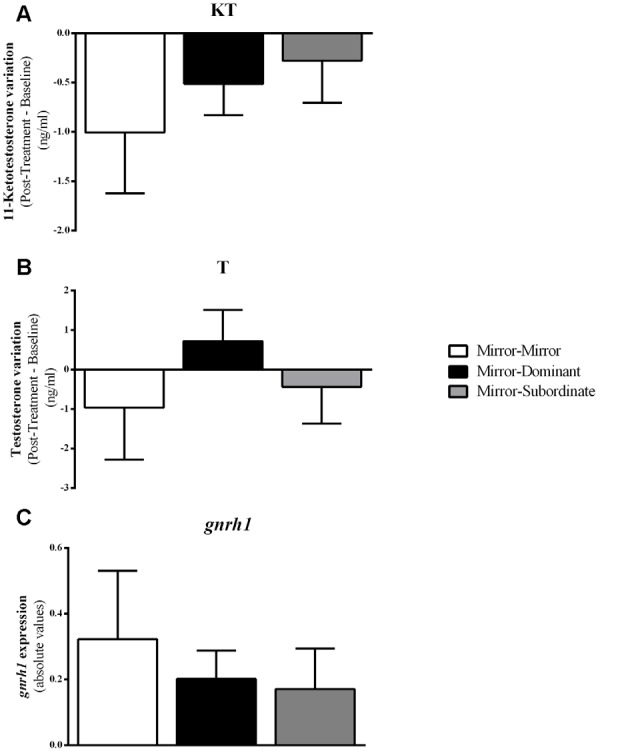
Variation in androgen levels and expression of gnrh1 in the Ppa of the focal fish for each experimental condition. **(A)** 11-Ketotestosterone (KT) levels; **(B)** Testosterone (T) levels; **(C)** *gnrh1* expression. Results are expressed as mean ± standard error of the mean (SEM).

Furthermore, there were no differences between treatments either in *gnrh1* expression in the PPa (*F*_(2,16)_ = 0.407, *p* = 0.672; MM vs. MD: *t*_(16)_ = 0.380, *p* = 0.704, *d* = 0.020; MM vs. MS: *t*_(16)_ = 0.903, *p* = 0.704, *d* = 0.053; MD vs. MS: *t*_(16)_ = 0.447, *p* = 0.704, *d* = 0.024), or in the androgen response to the behavioral treatment (KT: MM vs. MD: *t*_(12)_ = −0.644, *p* = 0.532, *d* = 0.041; MM vs. MS: *t*_(12)_ = −0.905, *p* = 0.383, *d* = 0.034; MD vs. MS: *t*_(10)_ = −0.441, *p* = 0.669, *d* = 0.006; T: MM vs. MD: *t*_(10)_= −0.984, *p* = 0.348, *d* = 0.306; MM vs. MS: *t*_(11)_= −0.377, *p* = 0.714, *d* = 0.034; MD vs. MS: *t*_(9)_ = 0.978, *p* = 0.353, *d* = 0.006; Figure 5).

## Discussion

Contrary to our predictions, fish assigned to the MM and the MD treatments showed similar behavioral patterns, that is, they equally fought aggressively their opponents, suggesting that the focal fish of the MD condition did not interpret a recently winning male as having a higher social status than itself, i.e., fish did not perceive the MD interaction as a defeat. In this context, it seems plausible that the visual signal presented was insufficient *per se* to communicate higher status, originating an agonistic interaction that, like the MM, was also unsolved, either because of the short interaction time allowed (only 2 min) or because of the symmetry of the fight. A study in another cichlid fish has shown that males previously interacting with a mirror have a higher probability to win a fight than non-mirror stimulated control individuals, probably because of an enhanced aggressive motivation (Dijkstra et al., 2012). On the other hand, the opponent fish had just won a fight, which is known to induce motivational changes that lead to the winner effect (Oliveira et al., 2009). Thus, it seems plausible that the behavior of the MD opponent was paralleled by that of the focal fish due to the heightened motivation of both contestants. In the case of the MS treatment, the losing experience of the opponent leads to a decrease in the willingness to engage in another contest (Hsu et al., 2006). So, it is plausible that the focal fish interpreted the interaction outcome as a win since they performed aggressive displays towards the subordinate opponent male first, which replied much later. Thus, due to a lack of an aggressive motivation by the opponent the focal fish did not further escalate its aggressive behavior (no attacks or bites), hence avoiding extra energetic costs (Hsu et al., 2011). Thus, at least for the MD condition, the experimental treatment may not have effectively altered the focal fish’s perception of the outcome, yet fish seem to constantly monitor the social interaction and adjust their behavior according to their internal state and to the behavior of their opponent. The ability of fish to compare their behavior with the one of the opponents and assess their competitive ability (mutual assessment) has few support in the literature (Hsu et al., 2011) but our data suggest its involvement. Of course, future experiments are necessary to fully uncover the underlying cognitive mechanisms.

In the present study, we showed that the pattern of expression of immediate early genes across the SDMN responds to acute changes in social interactions. Only 2 min of exposure to different fight outcomes (i.e., MD vs. MS) of an interaction that was already going on for 15 min was sufficient to trigger different patterns of *c-fos* and *egr-1* expression. Given the pivotal role of these immediate early genes in orchestrating integrated transcriptome changes (Clayton, 2000), these short-term responses of *c-fos* and *egr-1* to acute changes in the perceived dynamics of the interaction suggest that the neurogenomic state of the SDMN can change rapidly in response to perceived social interactions.

Our results also confirm the hypothesis, that the expression of social behavior is better explained by the overall pattern of activation of the SDMN rather than by the activity of a specific region in the brain (e.g., a specific node of the network; Teles et al., 2015). Indeed, there were no significant correlations between the expression of any of the immediate early genes tested and the expression of aggressive behavior. In contrast, the correlation matrices for the expression of each IEG across the nodes of the SDMN, which capture the co-activation or reciprocal inhibition between brain regions, were specific for each experimental treatment. Moreover, only the expression of *c-fos* in the TA was significantly different between experimental treatments (i.e., MM and MS treatments). The TA is the putative homolog of the ventromedial hypothalamus in mammals, and its ventrolateral subdivision has been strongly associated with aggression. For instance, pharmacogenetic inactivation of this area in mice stops inter-male aggressive behavior while optogenetic activation induces attacks towards females or inanimate objects (Lin et al., 2011). Other study analyzed the *c-fos* expression in the brain of subordinate hamsters after a fight and detected elevated activation in several areas including the lateral part of the ventromedial hypothalamus in comparison with dominant males (Kollack-Walker et al., 1997). In a recent review, Hashikawa et al. (2017) proposed the involvement of this particular sub-nucleus in the following aspects of aggression: aggressive motivation, specifically that the activation of this area heightens aggressive state (Falkner and Lin, 2014); detection of aggressive signals, such as for example olfactory cues (Falkner and Lin, 2014; Lin et al., 2011); and in the start and execution of aggressive behavioral patterns (Falkner and Lin, 2014). Our results only partially agree with this research in mammals since we report an accentuated expression of *c-fos* only in one of the two treatments (i.e., in MM but not in MD) in which fish express high levels of aggression and a decreased expression of this IEG when fish see a subordinate male after interacting with a mirror (MS) and consequently stop performing attacks and bites. In another cichlid fish (the Burton’s mouthbrooder, *Astatotilapia burtoni*) it has been demonstrated that males that were given an opportunity to rise in social rank have higher expression of *c-fos* and *egr-1* in all the areas of the SDMN, including the TA, when compared to stable males, either of a dominant or a subordinate social status (Maruska et al., 2013b). On the other hand, a social descending male has an increase of *c-fos*, and not *egr-1*, expression in this area (Maruska et al., 2013a), corroborating its involvement also in social status transitions, as observed in the current study.

Moreover, a very interesting finding was that fish that saw a subordinate male after fighting with a mirror (MS) showed an increase in the density of the structure of the SDMN, namely on the density of the *egr-1* network, when compared to the other treatments. This evidence suggests that the perception of the fight outcome (which only unequivocally occurred in this treatment) originated a denser brain network, which is characterized by redundant connections and hence a higher robustness to changes in its nodes (i.e., it is less likely affected by the removal of nodes at random (Makagon et al., 2012). Looking into centrality measures obtained with the network analysis it is possible to ascertain that the TA is a more central area while the PPa is a less important node of the *egr-1* network in the MM condition while in the MD and MS conditions the reversed pattern is observed. These results strengthen the idea of the main role of TA in status changes and of the PPa as a link to the bodily changes (e.g., androgen response) that should accompany the changes in brain state.

Androgens are known to respond to social interactions and this response has been hypothesized to play an adaptive role in the adjustment of aggressive behavior to the competitive demands of the social environment (challenge hypothesis, Hirschenhauser and Oliveira, 2006; Wingfield et al., 1990). Therefore, in this study, we have also investigated how androgens responded to the fighting assessment and how the changes in activation of the PPa, where GnRH1 neurons that control the HPG axis are located, were linked to a putative androgen response. Surprisingly, we found no significant changes in androgen levels in any of the treatments with social challenges (MD, MS). Concomitantly, we also did not find a change in the expression of gnrh1 in the PPa in response to the MD or MS treatments, and there were no correlations between gnrh1 expression and circulating androgen levels. Moreover, there were no correlations between the expression of any of the immediate early genes and that of *gnrh1*, indicating that the observed activation of the PPa in response to the experimental treatments does not correspond to an activation of the HPG axis. These negative results may result from the short time span of the staged fights with the real opponents, and/or from the failure to induce a perception of fight outcome in the case of the MD treatment.

In summary, our results support the view that it is the assessment that animals make of ongoing fights, and not the perception of the outcome, which triggers rapid changes in gene expression across the SDMN and that the TA is a key node in this network.

## Data Availability Statement

The datasets generated for this study are available on request to the corresponding author.

## Ethics Statement

The animal study was reviewed and approved by Direcção Geral de Alimentação e Veterinária, Lisbon, Portugal.

## Author Contributions

OA and RO designed the experiments. OA performed behavioral experiments. AF processed the samples. GO, JL and AF analyzed the data. AF, GO and RO wrote the article.

## Conflict of Interest

The authors declare that the research was conducted in the absence of any commercial or financial relationships that could be construed as a potential conflict of interest.
