# Supplementary material for: Fighting Assessment Triggers Rapid Changes in Activity of the Brain Social Decision-Making Network of Cichlid Fish
Source: Front Behav Neurosci. 2019 Sep 26;13:229. doi: 10.3389/fnbeh.2019.00229 (PMC6775253; doi:10.3389/fnbeh.2019.00229)
Supplement: Supplementary file 1 [file Data_Sheet_1.docx]

***Supplementary Material***

**

Figure S1.** Behavioral measurements for the focal fish - (A) frequency and (B) latency - and the opponent fish - (C) frequency and (D) latency - for each experimental condition.

**Table S1.** Primer sequences and annealing temperatures (Ta) for the genes studied.

| Gene | Primer sequences | Ta (ºC) |
| --- | --- | --- |
| *c-fos* | Fw: 5’-CCGTGGACACTCTGGGATA-3’  Rv: 5’-AAGGAGGCACTTGATGCTGT-3’ | 61 |
| *egr-1* | Fw: 5’-CTCTGGGCTGATAGGCAATGTT-3’  Rv: 5’-TGAGATGAGGACGAGGAGGTAGAA-3’ | 60 |
| *gnrh1* | Fw: 5’- TATCCTCAGAATGGCTGCAA -3’  Rv: 5’- GTTGTCCAGATCCCTCTTCC-3’ | 55 |
| *eef1A* | Fw: 5’- AGCAAGTACTACGTGACCATCATTG -3’  Rv: 5’- AGTCAGCCTGGGAGGTACCA -3’ | 61 |

*Fw: forward primer; Rv: reverse primer.*


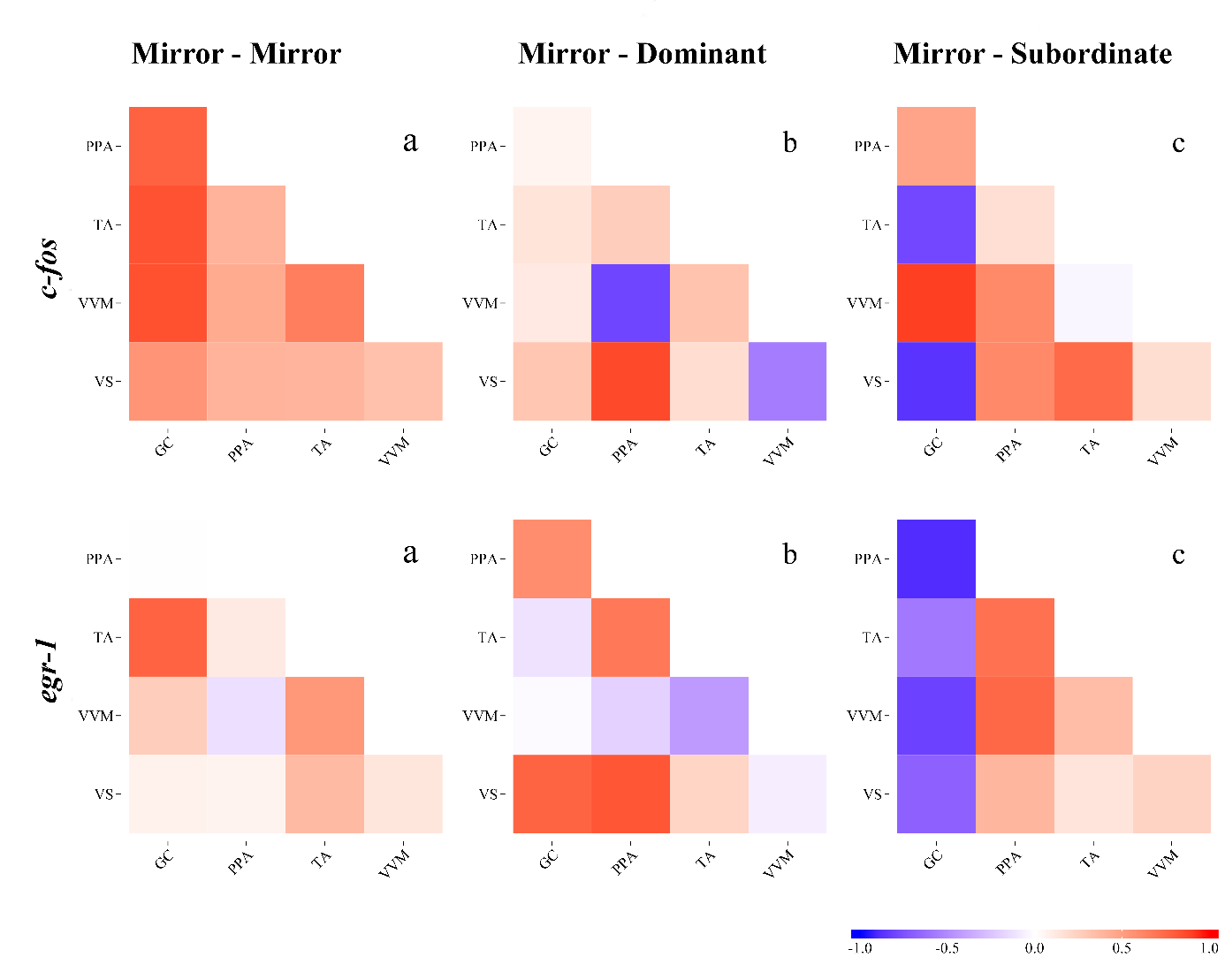
**Figure S2.** Functional connectivity in the SDMN network for all the experimental treatments, as measured by Pearson correlations between pairs of brain nuclei for *c-fos* and *egr-1*. Color scheme represents *r* values from −1 (blue) to 1 (red). GC, central gray; PPa, anterior part of the periventricular preoptic nucleus; TA, nucleus anterior tuberis; VVm, medial part of the ventral subdivision of the ventral telencephalon; Vs, supracommissural nucleus of the ventral telencephalon. Different letters indicate significantly different patterns of IEG expression in brain nuclei between treatments using the QAP correlation test.
